# Coexistence of *tet*(A) and *bla*_KPC-2_ in the ST11 hypervirulent tigecycline- and carbapenem-resistant *Klebsiella pneumoniae* isolated from a blood sample

**DOI:** 10.1007/s10096-022-04512-6

**Published:** 2022-11-02

**Authors:** Xiaokui Zhu, Changwu Yue, Huaixin Geng, Lingjie Song, Huiming Yuan, Xianqin Zhang, Chuanyu Sun, Guangxin Luan, Xu Jia

**Affiliations:** 1 Non-Coding RNA and Drug Discovery Key Laboratory of Sichuan Province, Chengdu Medical College, Chengdu, China; 2 Key Laboratory of Microbial Drugs Innovation and Transformation, Medical College, Yan’an University, Yan’an, China; 3 Huashan Hospital, Fudan University, Shanghai, China

**Keywords:** *Klebsiella pneumoniae*, Hypervirulent, Tigecycline-resistant, Carbapenem-resistant, ST11

## Abstract

Carbapenem-resistant *Klebsiella pneumoniae* are distributed worldwide. This study aimed to characterize a hypervirulent tigecycline-resistant and carbapenem-resistant *Klebsiella pneumoniae* strain, XJ-K2, collected from a patient’s blood. We tested antimicrobial susceptibility, virulence, and whole-genome sequencing (WGS) on strain XJ-K2. WGS data were used to identify virulence and resistance genes and to perform multilocus sequence typing (MLST) and phylogenetic analysis. Three novel plasmids, including a pLVPK-like virulence plasmid (pXJ-K2-p1) and two multiple resistance plasmids (pXJ-K2-KPC-2 and pXJ-K2-p3), were discovered in strain XJ-K2. The IncFII(pCRY) plasmid pXJ-K2-p3 carried the *dfrA14*, *sul2*, *qnrS1*, *bla*_LAP-2_, and *tet*(A) resistance genes. The IncFII(pHN7A8)/IncR plasmid pXJ-K2-KPC-2 also carried a range of resistance elements, containing *rmtB*, *bla*_KPC-2_, *bla*_TEM-1_, *bla*_CTX-M-65_, and *fosA3*. MLST analysis revealed that strain XJ-K2 belonged to sequence type 11 (ST11). Seven complete phage sequences and many virulence genes were found in strain XJ-K2. Meanwhile, antimicrobial susceptibility tests and *G. mellonella* larval infection models confirmed the extensively drug resistance (XDR) and hypervirulence of KJ-K2. To our knowledge, this is the first observation and description of the ST11 hypervirulent tigecycline- and carbapenem-resistant *K. pneumoniae* strain co-carrying *bla*_KPC-2_ and the *tet*(A) in a patient’s blood in China. Further investigation is needed to understand the resistance and virulence mechanisms of this significant hypervirulent tigecycline- and carbapenem-resistant strain.

## Introduction

*Klebsiella pneumoniae* is a common Gram-negative opportunistic pathogen that causes multiple diseases, including pneumonia, bacteremia, liver abscess, and urinary tract infections [1]. Carbapenem-resistant *K. pneumoniae* (CRKP) and hypervirulent *K. pneumoniae* (hvKP) have emerged as the two major types of clinically significant pathogens in China [2]. CRKP is commonly resistant to multiple antibiotics, including carbapenems, quinolones, and aminoglycosides [3]. These widespread CRKP isolates represent an antibiotic resistance threat of the highest priority, limiting therapeutic options for these multidrug resistance strains [4]. In China, the most common clinical CRKP isolates belong to sequence type (ST) 11, considered the most infectious clone responsible for the increasing prevalence of CRKP [5].

HvKP is associated with high morbidity and mortality because it causes life-threatening and community-acquired infections, including liver abscesses, endophthalmitis, pneumonia, and meningitis in healthy individuals [6]. HvKP was first reported in Taiwan in the mid-1980s and 1990s [7]. In the decades since then, hvKP has been reported globally [8]. These hypervirulent strains have a hypermucoviscous phenotype that is detectable as a positive “string test” result and a hypervirulent phenotype that is attributable to the presence of a large pLVPK-like virulence plasmid, harboring two capsular polysaccharide (CPS) regulator genes (*rmpA* and *rmpA2*) and several siderophore gene clusters [6].

Most hvKP strains are susceptible to commonly used antibacterial agents except ampicillin [9]. Recent studies have shown that hvKP strains can acquire a carbapenemase-encoding plasmid to transform into carbapenem-resistant hvKP (CR-hvKP) strains, and CRKP strains can evolve to become CR-hvKP strains by acquiring a pLVPK-like virulence plasmid [10]. These strains have made clinical treatment and infection control more difficult because they are simultaneously multidrug-resistant, hypervirulent, and highly transmissible [11]. Due to the emergence of CRKP strains, tigecycline was considered as a last-resort treatment for CRKP infections [12]. In recent years, however, some tigecycline- and carbapenem-resistant *K. pneumoniae* (TCRKP) have been reported, which has further limited the concern with respect to therapeutic selection [13]. However, until now, the mechanisms underlying *K. pneumoniae*’s resistance to tigecycline have not been fully understood. Previous studies showed that overexpression of the efflux pumps and alteration in the tigecycline target site played important roles in mediating tigecycline resistance [14].

In addition, recent research indicated that deletion of ramR and *tet*(A) is associated with resistance to tigecycline in *K. pneumoniae* [13]. Although Gu et al. reported the emergence of *bla*_KPC-2_ and the *tet*(A) in the ST11 hypervirulent TCRKP isolated from a patient’s gut, the occurrence of a TCRKP strain with these characteristics in a patient’s blood has not previously been reported [15]. This study reports a hypervirulent TCRKP strain, strain XJ-K2, isolated from a patient’s blood in China. To our knowledge, this is the first report of the ST11 hypervirulent tigecycline- and carbapenem-resistant *K. pneumoniae* strain co-carrying *bla*_KPC-2_ and *tet*(A) plasmids that have been detected in patient blood in China.

## Materials and methods

### Bacterial strain

In May 2018, *K. pneumoniae* strain XJ-K2 was isolated from a blood sample collected from a patient at a teaching hospital in Shanghai, China.

### Antimicrobial susceptibility testing

The broth microdilution method was used to determine the minimum inhibitory concentration (MIC) values of 27 antimicrobial agents. The positive control well (without antibiotics) showed significant bacterial growth and the MIC values could be read. The MICs were interpreted following the Clinical and Laboratory Standards Institute (CLSI, 2018). As there are no CLSI breakpoints for tigecycline and colistin, the European Committee on Antimicrobial Susceptibility Testing (EUCAST) followed the MIC interpretation. *Escherichia coli* ATCC 25,922 was used as quality control strain for antimicrobial susceptibility testing.

### Virulence testing for hypervirulent *K. pneumoniae *strain XJ-K2

A hypermucoviscous phenotype was determined using the string test previously described [6]. The *Galleria mellonella* (*G. mellonella*) model was used to evaluate the virulence of *K. pneumoniae* strains [3]. *G. mellonella* larvae (~ 300 mg; Tianjin Huiyude Biotech Company, Tianjin, China) were used to test the virulence of the strain XJ-K2. Each group selected ten randomly selected insects weighing 250–350 mg. Overnight cultures of strains XJ-K2, WCHKP030925 (hypervirulent control), and WCHKP13F4 (low virulence control) were washed with phosphate-buffered saline (PBS) and further adjusted with PBS to concentrations of 1 × 10^5^, 1 × 10^6^, and 1 × 10^7^ CFU/ml [16]. Ten microliters of inoculum was injected into *G. mellonella* larvae through the last left pro-leg, followed by incubation at 37 ℃ in darkness [3]. The number of dead larvae was counted at 12-h intervals up to 72 h after the incubation.

### WGS and bioinformatics analysis

Illumina HiSeq 4000 and the PacBio RS II platform were used to sequence the genome of strain XJ-K2 at the Beijing Genomics Institute. The program Pbdagcon (https://github.com/PacificBiosciences/pbdagcon) was used for self-correction [17]. Draft genomic unitigs were assembled using the Celera Assembler against a high-quality corrected circular consensus sequence subreads set [18]. GATK (https://www.broadinstitute.org/gatk/) and SOAP tool packages were used to make single-base corrections [19]. RAST 2.0 (https://rast.nmpdr.org/), Prokka (http://vicbioinformatics.com/), and BLASTN (https://www.ncbi.nlm.nih.gov/) were used to annotate the genome sequence of strain XJ-K2. ORFfinder (https://www.ncbi.nlm.nih.gov/orffinder/) was used to predict the open reading frames (ORFs). Annotation of mobile elements, drug resistance genes, and other characteristic features was performed by IS*finder*, Resfinder 3.1, and INTEGRALL. For the classification of plasmids, PlasmidFinder 2.0 was used [20]. The MLST of *Klebsiella pneumoniae* strain XJ-K2 was performed using MLST 2.0 (https://cge.cbs.dtu.dk/services/MLST/). A comparative plasmid map was generated by the BLAST Ring Image Generator (BRIG). Gene comparative structure diagrams were created in Easyfig. The nucleotide sequence accession numbers for *K. pneumoniae* strain XJ-K2 in GenBank are CP032240 (chromosome), CP032241 (pXJ-K2-p1), CP032242 (pXJ-K2-KPC-2), and CP032243 (pXJ-K2-p3).

### Phylogenetic analysis

In this study, Unipro UGENE 1.32 (http://ugene.net/) was used to perform phylogenetic analysis. We selected the genome sequences of representative *K. pneumoniae* strains that carry a pLVPK-like virulence plasmid to construct a phylogenetic tree for *K. pneumoniae* strain XJ-K2.

## Results

### Isolation of a hypermucoviscous multiresistant *K. pneumoniae* strain

*Klebsiella pneumoniae* XJ-K2 was isolated from the Intensive Care Unit (ICU) of Huashan Hospital of Fudan University in Shanghai in May 2018. It was derived from a blood sample of a patient. The patient was admitted to the ICU with cerebral hemorrhage and was treated with indwelling catheterization. The urinary tract infection combined with bacteremia could be a risk factor for the patient’s poor prognosis. Antimicrobial susceptibility testing showed that strain XJ-K2 was extensively drug-resistant (XDR). Therefore, the patient was treated with polymyxin B 120 mg/day. After 1-week follow-up, no obvious abnormalities were found in the blood culture, and clinical symptoms were obviously improved. XDR-*K. pneumoniae* is considered a huge health concern due to the few treatment options available in the clinic. XJ-K2 was determined as a hypermucoviscous strain by the “string test.” Hypermucoviscous *K. pneumoniae* was diagnosed by a positive string test (> 5 mm) [6].

**Fig. 1:**
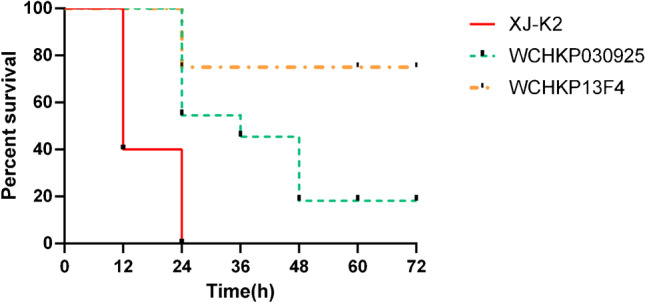
Survival (%) over time of *Galleria mellonella* infected with *Klebsiella pneumoniae* strains. For each strain, 1 × 10^7^ CFU/ml was used to infect *G. mellonella*. The survival of *G. mellonella* was measured every 24 h. *K. pneumoniae* WCHKP030925 and WCHKP13F4 were the hypervirulent and low virulence controls, respectively

The *G. mellonella* model was used to evaluate the virulence of *K. pneumoniae* strains in vivo. *G. mellonella* was infected by *K. pneumoniae* strains through exposure to inoculum concentrations of 1 × 10^7^ CFU/ml for 48 h, and the survival rates of *G. mellonella* infected by strains XJ-K2, WCHKP030925, and WCHKP13F4 were 0.0%, 10.0%, and 60%, respectively (Fig. 1). Based on these results, strain XJ-K2 was identified as hypervirulent *K. pneumoniae*.

The broth microdilution method was used to determine the minimum inhibitory concentration (MIC) of 27 antimicrobial agents. The results showed that strain XJ-K2 was extensively drug-resistant (XDR), and high-level resistance was observed against broad-spectrum cephalosporins**,** aminoglycosides, fosfomycin, and folate pathway inhibitors (Table 1). Of note, MIC > 2 μg/ml of polymyxin B in *K. pneumoniae* was regarded as being resistant to polymyxin B according to EUCAST. In this study, the MIC of polymyxin B was 2 μg/ml, close to the critical value of polymyxin B resistance, which might be a potential threat to clinical treatment.

**Table 1:** Resistance of *Klebsiella pneumoniae* strain XJ-K2 against antimicrobial agents

Antimicrobial agents	MIC (μg/ml)	Interpretation
Piperacillin	> 1024	R
Ampicillin	> 1024	R
Ceftazidime	512	R
Ceftriaxone	> 1024	R
Cefotetan	1024	R
Cefuroxime	> 1024	R
Cefazolin	> 1024	R
Cefoxitin	512	R
Meropenem	128	R
Imipenem	32	R
Aztreonam	> 1024	R
Tobramycin	> 1024	R
Gentamicin	> 1024	R
Amikacin	> 1024	R
Kanamycin	> 1024	R
Netilmicin	> 1024	R
Ciprofloxacin	64	R
Levofloxacin	64	R
Doxycycline	128	R
Minocycline	64	R
Tetracycline	256	R
Tigecycline	4	R
Fosfomycin	1024	R
Nitrofurantoin	256	R
Trimethoprim	> 1024	R
Sulfamethoxazole	> 1024	R
Polymyxin B	2	S

*MIC* minimum inhibitory concentration, *R* resistant, *I* intermediate, *S* susceptible

### Genome features and chromosome analysis

**Fig. 2:**
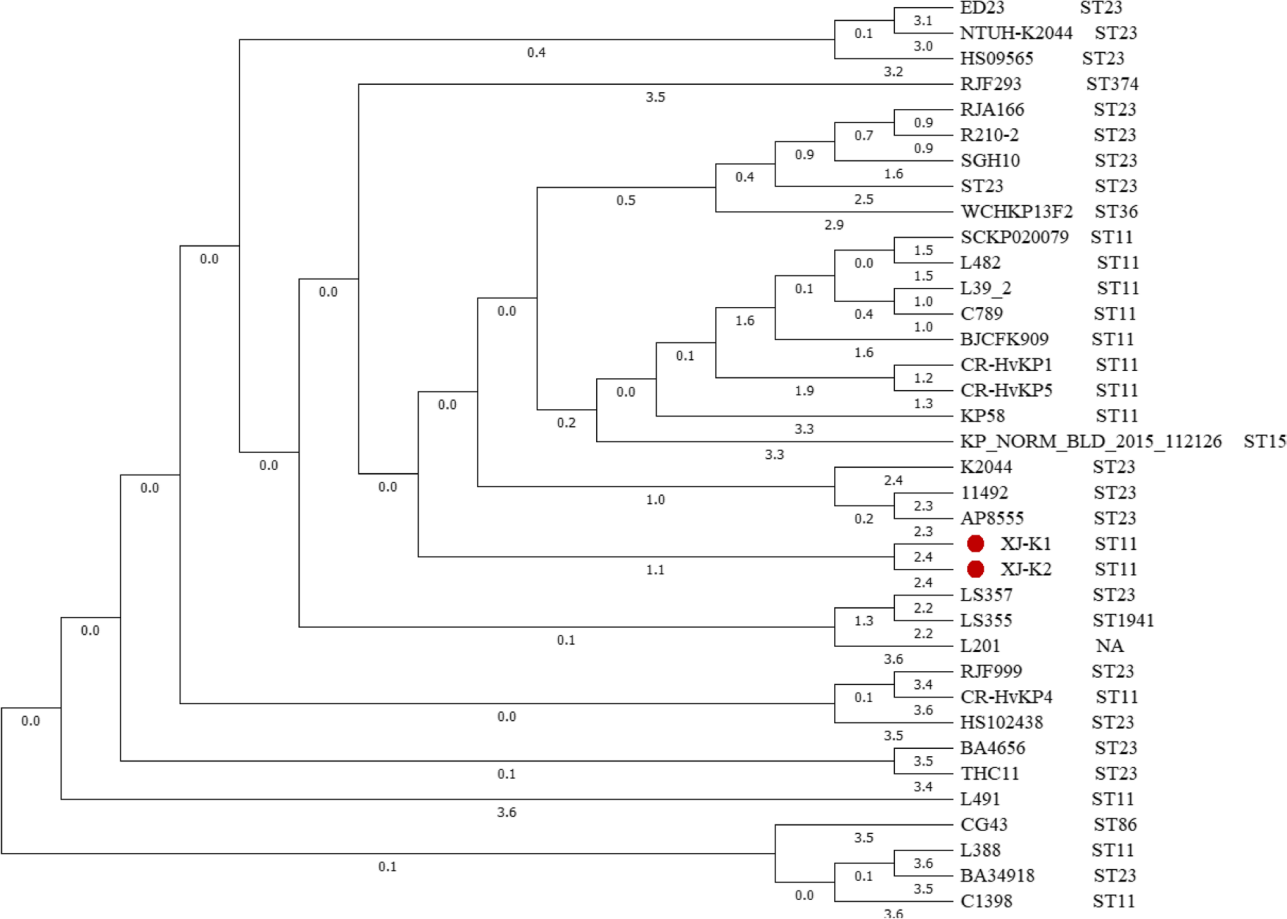
Phylogenetic tree of *Klebsiella pneumoniae* strains. The tree was constructed using Unipro UGENE 1.32. High genetic relatedness was observed between *K. pneumoniae* strains XJ-K2 and XJ-K1 (CP032163)

The results of our analysis showed that the genome of strain XJ-K2 contains a chromosome (5,503,278 bp) and three plasmids: pXJ-K2-p1 (219,775 bp), pXJ-K2-KPC-2 (96,030 bp), and pXJ-K2-p3 (84,855 bp; Table 2). The final draft genome revealed that the strain XJ-K2 chromosome contains 57.31% G + C content and 5715 predicted ORFs. Twelve antibiotic resistance genes were identified in strain XJ-K2, and the resistance genes *fosA*, *mdf(A)*, and *blaSHV-182* were located on the chromosome. Furthermore, among genes located on the strain XJ-K2 chromosome, *gyrA* had two sites with amino acid mutations (Ser83Ile and Asp87Gly) and *parC* had one (Ser80Ile) [21]; these mutations are associated with quinolone resistance. The strain XJ-K2 carried four genes encoding β-lactamases, one of which was a carbapenemase gene (*bla*_*KPC-2*_; Table 3). Our analysis showed that strain XJ-K2 had several virulence factors, including the regulators of capsular polysaccharides (*rmpA* and *rmpA2*), the type 1 fimbriae fimbriae-encoding system (*fimABCDEFGHIK*), the type 3 fimbriae-encoding system (*mrkABCDF*), aerobactin (*iucABCD* and *iutA*), and yersiniabactin (*fyuA*, *irp1*, *irp2*, and *ybtAEPQSTUX*). MLST analysis showed that strain XJ-K2 belonged to sequence type 11. The phylogenetic analysis showed that the ST11 hvKP strain XJ-K2 was highly similar to the hvKP strain XJ-K1 (Fig. 2). Furthermore, seven complete phage sequences, ranging in size from 37.5 to 59.6 kb, were found in strain XJ-K2 (Table 4).

**Table 2:** Major features of the *Klebsiella pneumoniae* strain XJ-K2 genome

Category	Chromosome	pXJ-K2-p1	pXJ-K2-KPC-2	pXJ-K2-p3
Size (bp)	5,503,278	219,775	96,030	84,855
G + C (%)	57.31	50.02	53.81	54.10
Predicted ORFs	5715	253	177	161
Resistance gene (s)	*fosA*, *mdf(A)*, *bla*_SHV-182_, ramR	None	*rmtB*, *bla*_KPC-2_, *bla*_TEM-1_, *fosA3*, *bla*_CTX-M-65_	*sul2*, *qnrS1*, *dfrA14, bla*_LAP-2_, *tet*(A)

**Table 3:** Resistance genes in *Klebsiella pneumoniae* strain XJ-K2

Genome	Gene	Function	Gene cassette	Integron
Chromosome	*mdf(A)*	Macrolide resistance	−	ND
	*bla*_SHV-182_	β-Lactam resistance	−	ND
	*fosA*	Fosfomycin resistance	−	ND
pXJ-K2-KPC-2	*rmtB*	Aminoglycoside resistance	−	ND
	*bla*_TEM-1_	β-Lactam resistance	−	ND
	*fosA3*	Fosfomycin resistance	−	ND
	*bla*_CTX-M-65_	β-Lactam resistance	−	ND
	*bla*_SHV-12_	β-Lactam resistance	−	ND
	*bla*_KPC-2_	β-Lactam resistance	−	ND
pXJ-K2-p3	*dfrA14*	Trimethoprim resistance	+	Class 1 integron
	*sul2*	Sulfonamide resistance	−	ND
	*qnrS1*	Quinolone resistance	−	ND
	*bla*_LAP-2_	*β*-lactam resistance	−	ND
	*tet(*A)	Tigecycline resistance	−	ND

+ gene was located within a gene cassette, − gene was not located within a gene cassette, *ND* gene cassette was not related to an integron

**Table 4:** Phage sequences in *Klebsiella pneumoniae* strain XJ-K2

Location	Phage start	Phage end	Phage length	GC
Chromosome	1,215,753	1,254,511	38,759	53.72%
Chromosome	1,359,257	1,401,804	42,548	56.28%
Chromosome	1,529,795	1,574,986	45,192	50.35%
Chromosome	3,085,091	3,144,733	59,643	52.66%
Chromosome	3,329,233	3,369,548	40,316	54.00%
Chromosome	3,385,576	3,429,904	44,329	50.35%
Chromosome	3,702,927	3,740,426	37,500	51.11%

### Sequence analysis of two multidrug-resistant plasmids

In this study, the plasmids harboring *bla*_*KPC-2*_ and the *tet(A)* were designated pXJ-K2-KPC-2 and pXJ-K2-p3, respectively. Plasmids pXJ-K2-KPC-2 and pXJ-K2-p3 belonged to the IncFII(pHN7A8)/IncR and IncFII(pCRY) groups. Both plasmids contained genes conferred antibiotic resistance, conjugal transfer, replication, and stability.

**Fig. 3:**
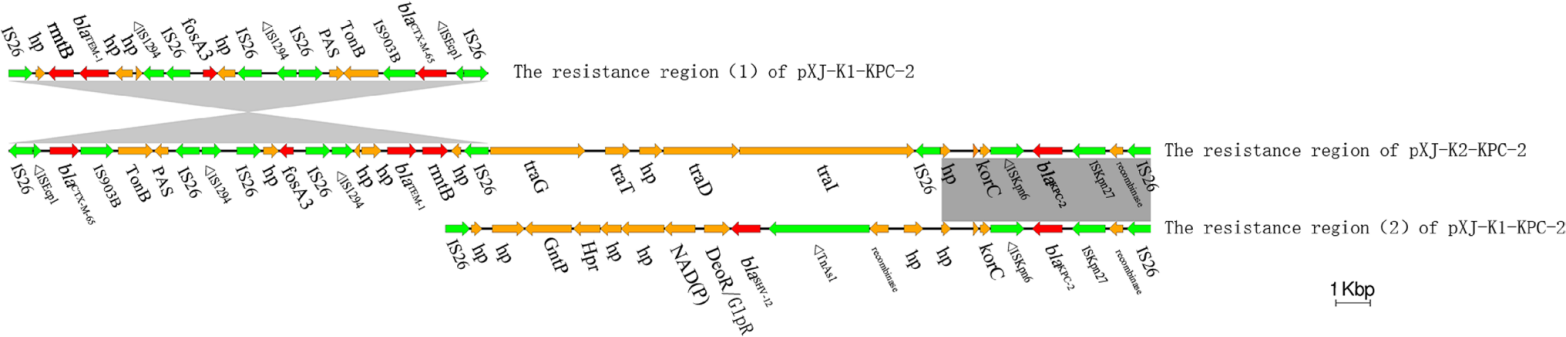
Resistance region of pXJ-K2-KPC-2 and comparison with related regions. Genes are denoted by arrows. Resistance genes, mobile elements, and other features are colored based on their functional classification. Shading denotes regions of homology (> 95% nucleotide identity)

Sequence alignments using BLASTN showed that pXJ-K2-KPC-2 shared 79% query coverage and 99% identity with plasmid unnamed3, as described in our previous report (GenBank accession number CP032166). The resistance region of pXJ-K2-KPC-2 contained three resistance units (IS*26-*ΔIS*Ecp1*-*bla*_*CTX*-*M*-*65*_-IS*903B*, IS*26*-*fosA3*-IS*26-*ΔIS*1294*-*bla*_*TEM-1*_*-rmtB1-*IS*26*, and IS*26-*ΔIS*kpn6*-*bla*_*KPC*-*2*_*-*IS*kpn27*-IS*26*) (Fig. 3). The three resistance units conferred resistance to aminoglycoside, carbapenems, β-lactam, and fosfomycin.

**Fig. 4:**
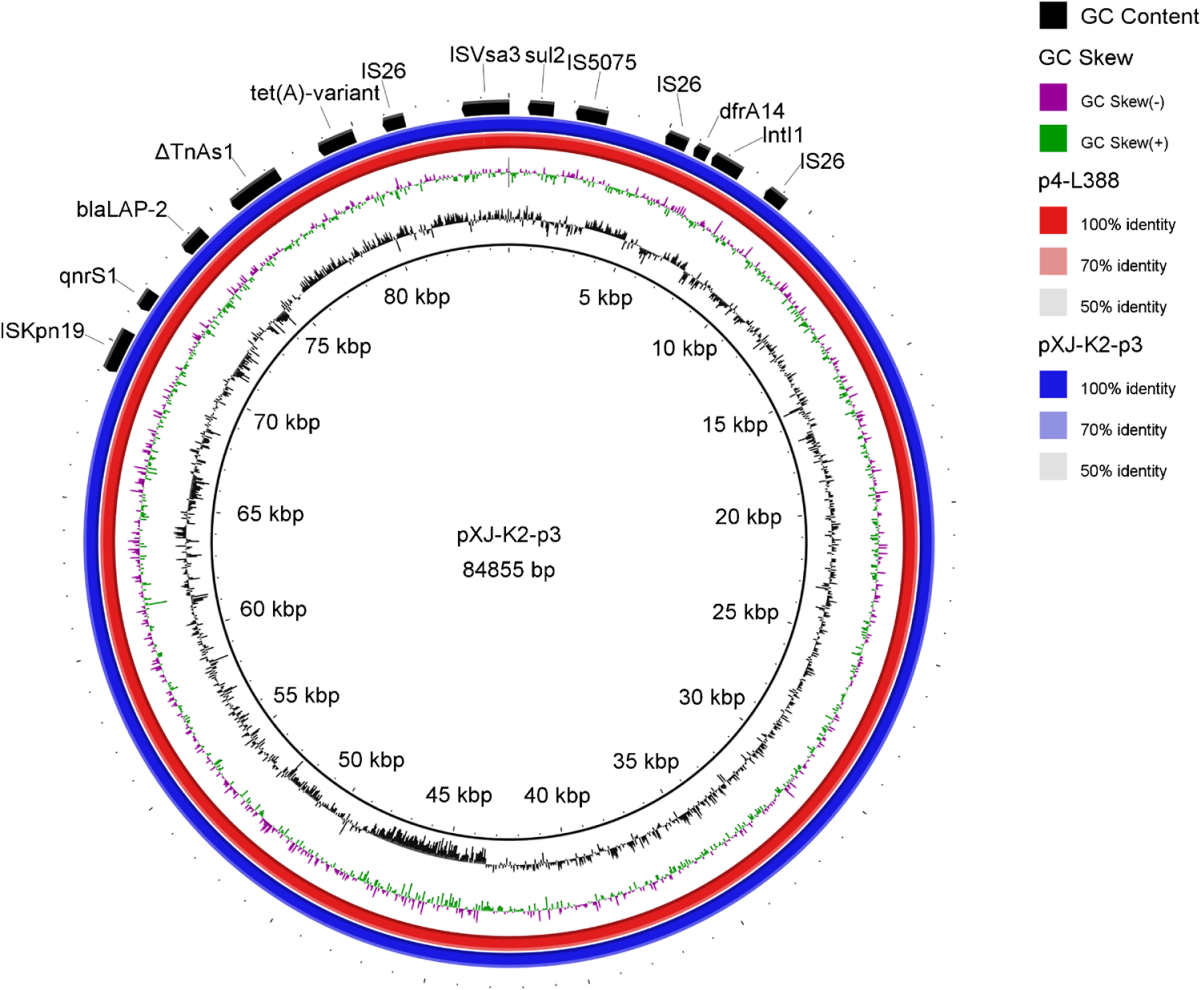
A comparative plasmid map of pXJ-K2-p3 and p4-L388 (CP029223) was drawn with BLAST Ring Image Generator by using pXJ-K2-p3 as a reference. A sequence comparison revealed that pXJ-K2-p3 showed 100% query coverage and 99.97% identity with p-L388

The plasmid pXJ-K2-p3 was highly similar (100% query coverage, 99.97% identity) to the plasmid p4-L388 (GenBank accession number CP029223). These plasmids harbored two resistance modules, namely, the dfrA14 region and the *tet*(A) region (Fig. 4). The dfrA14 region consisted of two resistance units, IS*26-IntI1-dfrA14-*IS*26* and IS*Vsa3-sul2-*IS*5075*. Resistance gene dfrA14 was located within a gene cassette related to In191 (Table 3). Moreover, the *tet*(A) region contained the resistance genes *qnrS1*, *bla*_*LAP-2*_, and *tet*(A), which conferred resistance to quinolone, β-lactam, and tigecycline.

### Sequence analysis of the pLVPK-like virulence plasmid

**Fig. 5:**
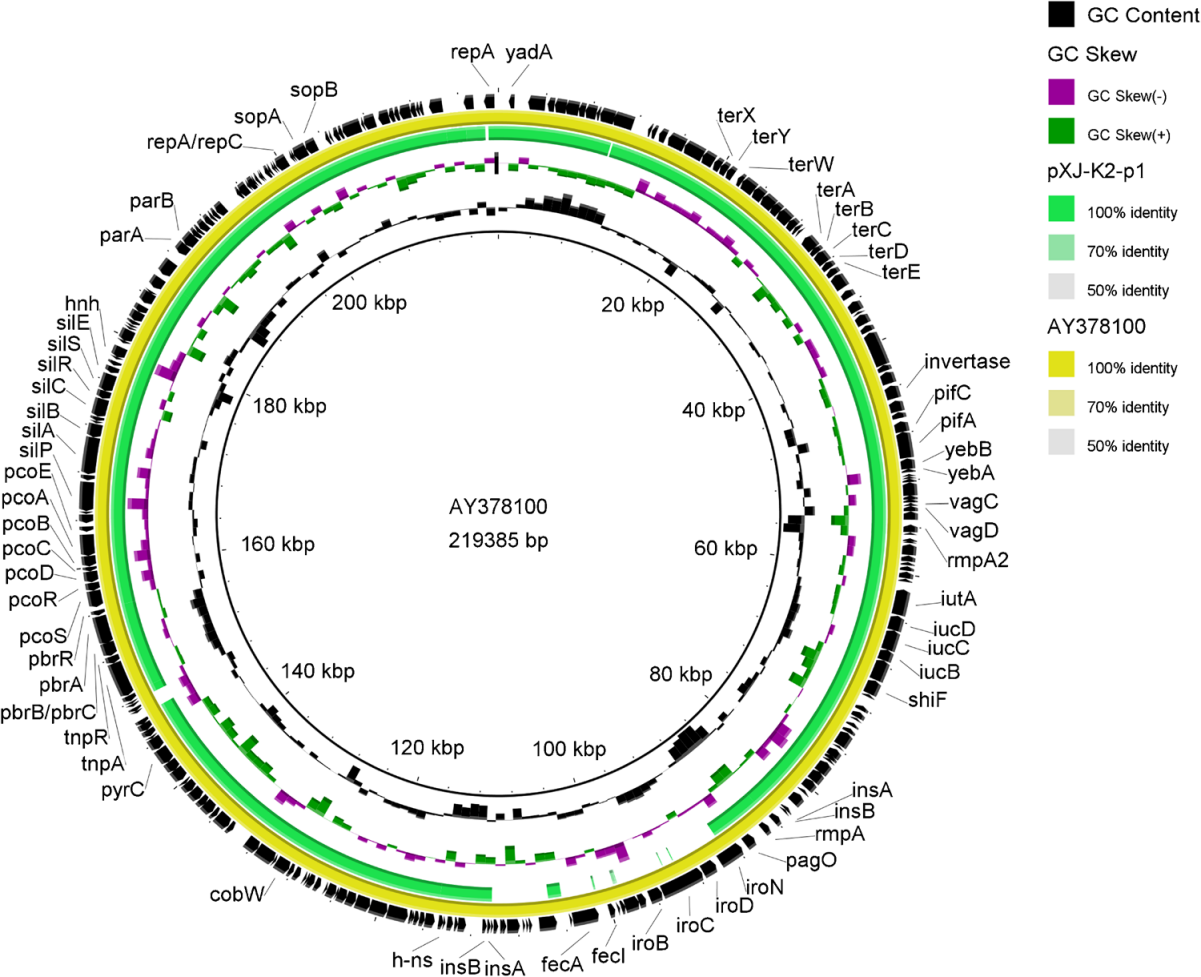
Comparative plasmid map comparing typical virulence plasmid pLVPK (AY378100) with the pLVPK-like virulence plasmid pXJ-K2-p1 from strain XJ-K2. The map was drawn with BLAST Ring Image Generator by using pLVPK as a reference. Sequence alignment revealed that pXJ-K2-p1 showed 90% query coverage and 99.06% identity with pLVPK

The IncHI1B plasmid pXJ-K2-p1 (50.02% G + C content, 253 ORFs) was a virulence plasmid that contained the hvKP-associated virulence genes *iucABCD*, *rmpA*, *rmpA2*, and *iutA*. Nevertheless, no antibiotic-resistance genes were found on pXJ-K2-p1. Sequence alignments revealed that pXJ-K2-p1 exhibited 90% query coverage and 99.06% identity with the typical virulence plasmid pLVPK (GenBank accession number AY378100). Therefore, pXJ-K2-p1 was considered to be a pLVPK-like virulence plasmid. To understand the difference in virulence genes between the pXJ-K2-p1 and pLVPK virulence plasmids, we constructed a comparative plasmid map, comparing the typical virulence plasmid pLVPK (AY378100) with the plasmid pXJ-K2-p1 using BLAST Ring Image Generator (Fig. 5). As shown in Fig. 5, genes *iroBCD*, *fecA*, *fecR*, and *fecl* were deleted in the pXJ-K2-p1 plasmid.

## Discussion

CR-hvKP strains have been identified in multiple sequence types, including ST23, ST11, ST36, and ST65 [22]. Gu et al. reported that some ST11 CRKP strains transform into CR-hvKP by obtaining a pLVPK-like virulence plasmid [10]. In addition, Liu et al. reported that ST23 CR-hvKP emerged following its acquisition of carbapenemase plasmids [23], suggesting that the horizontal transfer of virulence plasmids into CRKP strains and carbapenemase plasmids into hvKP strains has substantially contributed to the emergence of multiple sequence types of CR-hvKP. In this study, strain XJ-K2 was resistant to tigecycline, meropenem, and imipenem (Table 1), which suggests that strain XJ-K2 is a TCRKP. The ST11 hypervirulent TCRKP strain XJ-K2 revealed two typical features of hvKP, i.e., a hypermucoviscous phenotype and a pLVPK-like virulence plasmid encoding the virulence genes *rmpA*, *rmpA2*, *iucABCD*, and *iutA*. Hypervirulent TCRKP in patient blood limits therapeutic options and poses a huge threat to public health. Analysis of its genome sequence revealed that multiple resistance determinants (MDRs) were present in the ST11 hypervirulent TCRKP strain (Table 4), which is the first report of the first time that an ST11 hypervirulent TCRKP strain co-producing *bla*_*KPC-2*_ and the *tet*(A) has been isolated from patient blood in China.

Phylogenetic analysis of strain XJ-K2 revealed that it is closely related to strain XJ-K1 (CP032163, ST11 and Shanghai, China), suggesting that they share a common ancestor. One virulence plasmid (pXJ-K2-p1) and two MDR plasmids (pXJ-K2-KPC-2 and pXJ-K2-p3) were identified in the hypervirulent TCRKP strain XJ-K2. The plasmid pXJ-K2-p1 was highly similar to the typical virulence plasmid pLVPK, which was found in hvKP strain CG43. These two plasmids carried aerobactin-related genes (*iucABCD* and *iutA*) and capsular polysaccharide regulator genes (*rmpA* and *rmpA2*), which are characteristic features of hvKP [24]. Many virulence genes might have helped this strain to grow and replicate during host infection.

The MDR plasmid pXJ-K2-KPC-2 was similar to the plasmid unnamed3 (GenBank accession number CP032166). These two plasmids possessed IncFII(pHN7A8)/IncR backbones. Unlike the plasmid unnamed3’s resistance regions, the resistance unit IS*26*-*bla*_*SHV*-*12*_-*ΔTnAs1* was absent in pXJ-K2-KPC-2. These results indicated that plasmids with similar backbones could carry different resistance determinants that effectively confer resistance. In pXJ-K2-KPC-2, blaKPC-2 was associated with the IS*26*-based composite transposon, containing the ΔIS*Kpn6*-*bla*_*KPC*-*2*_-IS*Kpn27* core structure, which is the most common element in the genetic environment of blaKPC-2 in China [25].

The other MDR plasmid, pXJ-K2-p3, carried an IncFII(pCRY) backbone with five resistance determinants: *dfrA14*, *sul2*, *qnrS1*, *bla*_*LAP-2*_, and the *tet*(A). Furthermore, a total of seven complete phage sequences were detected in the strain XJ-K2 chromosome that contributed to the transfer of resistance genes and virulence factors [26]. As previously reported, there was a slight increase in the MIC value of tigatin in isolates carrying *tet*(A) [27]. Our study integrated the *tet*(A) was integrated into pXJ-K2-p3 via the insertion sequences IS26 and ΔTnAs1. The high mobility of IS26 could mediate the horizontal transmission of resistance genes or the functional deficiency of partial genes, which could be related to the genetic adaptation of plasmids under antibiotic pressure. Inserted tetA resulted in low levels of tigecycline resistance, which may be related to efflux mechanisms [28]. Interestingly, ramR gene was found on the chromosome of the XJ-K2 strain, which might be meaningful regarding increased resistance. Therefore, we speculated that *K. pneumoniae* carrying ramR and *tet*(A) tended to evolve tigecycline resistance under selective pressure more easily. However, it remains unknown whether tet(A) played a major role in tigecycline resistance, which needs further study. In191, from the dfrA14 region, IntI1, and dfrA14 were integrated into the plasmid via insertion sequence IS26. In the plasmid pKF3-140 [29], class 1 integron harbored three resistance genes, specifically *dfrA17*, *aadA5*, and *sul1*, which are associated with resistance to trimethoprim, aminoglycoside, and sulfonamide, suggesting that class 1 integron is vital in increasing multidrug resistance among bacterial strains [30].

## Conclusion

In conclusion, we have reported an ST11 hypervirulent TCRKP strain, XJ-K2, isolated from patient blood. We characterized the genomic features of strain XJ-K2 via WGS and bioinformatic analysis. To our best knowledge, this is the first report of the ST11 hypervirulent TCRKP strain co-carrying *bla*_*KPC-2*_ and the *tet*(A) has been isolated from patient blood in China. In addition, we should be devoted to concerning the spread of tet(A) and ramR in *K. pneumoniae* clinical isolates, which may be potential threats leading to tigecycline resistance. Therefore, active surveillance of this and other hypervirulent TCRKP strains and the development of new drugs are required to control the growth of these strains and prevent their transmission in hospitals.

## Data Availability

We have uploaded the sequencing data of the article to the repository (https://www.ncbi.nlm.nih.gov/). Additional data are presented in this manuscript in the main text.
